# Impact of habitat alteration on amphibian diversity and species composition in a lowland tropical rainforest in Northeastern Leyte, Philippines

**DOI:** 10.1038/s41598-020-67512-6

**Published:** 2020-06-29

**Authors:** Syrus Cesar Pacle Decena, Carlo Aguirre Avorque, Ian Christopher Pacle Decena, Pol Delbert Asis, Bryan Pacle

**Affiliations:** Environmental Management Department, Visayas State University-Alangalang, Brgy. Binongto-an, 6517 Alangalang, Leyte, Philippines

**Keywords:** Forest ecology, Environmental impact

## Abstract

The impact of anthropogenic habitat alteration on amphibians was investigated, employing an investigative focus on leaf-litter and semi-aquatic species across different habitat alteration types. The habitat alteration types which include primary forest, selectively logged primary forest, secondary forest, abandoned farm areas and pasture (this represents a gradient of habitat alteration ranging from least altered to most altered, respectively) also encompass two habitat types: stream and terrestrial. Species assemblage was compared between habitat alteration types and habitat types, where a total 360 leaf-litter and semi-aquatic amphibians were observed (15 species, 6 families). It was found that amphibian abundance was significantly higher in both forest and stream habitat, and species richness did not differ with respect to habitat alteration type. It was determined, however, that species richness was highly dependent on habitat type (significantly higher in stream habitat). Meanwhile, diversity (Shannon–Wiener) was significantly higher in both forest and stream habitat, and species composition differed markedly between habitat alteration types for stream strip plots. Forest habitat exhibited domination by forest specialist species, while altered habitat (abandoned farm areas and pasture) exhibited domination by open-habitat specialist species. Additionally, strong relationships were found between species composition and abundance, as well as richness and diversity (within the measured habitat structures and observed microclimatic conditions). Analyses determined that the higher abundance of leaf-litter and semi-aquatic amphibians was best explained by higher DBH (1.3 m from the ground) and lower temperature and the higher species richness was best explained by higher understorey density. Additionally, higher diversity was associated with increasing understorey density, tree density and temperature. In general, the assemblage of leaf-litter and semi-aquatic amphibians in the lowland tropical rainforest in northeastern Leyte was affected by habitat alteration, highlighting the on-going importance of conservation efforts.

## Introduction

Though the Philippines is commonly known as one of the most important biodiversity hotspots on Earth^1^, there is undoubtedly cause for concern, since southeast Asia has the highest relative rate of deforestation of any major tropical region^2^. In the Philippines, an average of 162,000 ha of forests are cleared per year^3^ and consequently, the remaining primary forest of the country represents approximately only 3%^1^ of land area. This decline of the country's forest is commonly attributed to logging, urbanization and agricultural expansion, as well as other peripheral factors such as shifting cultivation, unorganized encroachment on forest lands, squatting, migration to upland forested areas, and government-sponsored settlement schemes^3,4^. Deforestation due to these human-induced activities adversely affects biodiversity, which in turn, is expected to result in species decline and an increase in species extirpation/extinction^5^.

Anthropogenic habitat alterations such as deforestation, fragmentation and conversion generally result in biodiversity loss^6^. For amphibians, and particularly for arboreal species, the reduction of suitable habitat has obvious and disconcerting links to species decline^7^. Previous studies have examined some of the effects of disturbance on amphibian communities, such as Cruz-Elizalde et al.^8^ which found a reduced number of species in disturbed habitats versus preserved habitats. In terms of amphibian species assemblage, generalist species tend to dominate in disturbed habitat, while undisturbed habitat tends to have a higher proportion of species with conservation concern. Similarly, the findings of Jongsma et al.^9^ indicate that diversity and richness were lower in secondary than they were in primary forest, suggesting that amphibian assemblages in interior forest habitat were more vulnerable to habitat alterations. In the Philippines, local extinction of amphibians has been attributed to the removal of large trees (primarily dipterocarps) and the resultant fragmentation and destruction of original forest^10,11^. This type of habitat alteration has important implications for forest-dwelling species such as amphibians, specifically where tropical rainforest continues to degrade and contract in size^11^. As a measure of resistance to threatening anthropogenic activity, amphibians, however, may be able to tolerate some habitat alterations as long as a high percentage of forest is retained^12^.

Habitat alteration is also associated with changes in habitat features which are also likely to influence amphibian diversity^9^. Habitat features and environmental factors that typically influence amphibian diversity and distribution are canopy cover, leaf-litter cover, understory density, temperature and rainfall, elevational gradient, forest patch and landscape attributes^13–16^. For example, climatic factors such as temperature and precipitation are known to influence breeding performance, morphology, developmental rate and survival of amphibians. Specifically, warming temperature increased or decreased body size and rainy periods increased or reduced the survival of amphibian species^17^.

The Philippine archipelago is home to more than 100 amphibians^10^, though this diversity is under the constant threat of habitat alteration. In spite of the relatively high diversity and endemism of amphibians in the Philippines, the current understanding on the response of these organisms to habitat modification and alteration remains limited. Most existing studies on Philippine amphibians focus on taxonomic surveys^18–25^, and distribution and endemism^26,27^. Conversely, very few existing research efforts focus on the impact of habitat alteration and fragmentation on amphibians^11^. Thus, this study aims to evaluate the response of amphibians to habitat alteration, particularly the leaf-litter and semi-aquatic species, and to examine relationships between environmental variables. Specifically, the objectives of the study are (a) to determine any differences in abundance, richness and diversity (Shannon–Wiener) of leaf-litter and semi-aquatic amphibians among habitat types (stream and terrestrial) in gradient of habitat alteration (primary forest, selectively logged primary forest, secondary forest, abandoned farm areas and pasture); (b) to examine how species composition varies between habitat alteration types; (c) to examine how habitat characteristics influence the pattern of distribution of leaf-litter and semi-aquatic amphibians; and (d) to determine which environmental variables influence the abundance, richness and diversity of leaf-litter and semi-aquatic amphibians.

## Results

### Differences and correlation among environmental variables

The environmental variables recorded among habitat alteration types for both stream and terrestrial habitat are presented in Table 1. These environmental variables differed significantly between habitat alteration types. Most of the variables were lower in abandoned farm areas and pasture compared to forest habitats for stream habitats, including understorey density (H = 20.533, DF = 4, *P* ≤ 0.0001), elevation (H = 22.646, DF = 4, *P* ≤ 0.0001), leaf litter thickness (H = 20.882, DF = 4, *P* ≤ 0.0001), leaf litter volume (H = 17.592, DF = 4, *P* = 0.001), DBH (1.3 m from the ground) (H = 19.520, DF = 4, *P* = 0.001), tree height (H = 24.679, DF = 4, *P* ≤ 0.0001) and tree density (H = 18.753, DF = 4, *P* = 0.001). Likewise, the same trend was observed for terrestrial habitats with the same environmental variables being lower in abandoned farm areas and pasture compared to forest habitats; understorey density (H = 26.123 , DF = 4, *P* ≤ 0.0001), elevation (H = 27.085, DF = 4, *P* ≤ 0.0001), leaf litter thickness (H = 27.306, DF = 4, *P* ≤ 0.0001), leaf litter volume (H = 27.066, DF = 4, *P* ≤ 0.0001), DBH (H = 25.553, DF = 4, *P* ≤ 0.0001), tree height (H = 26.947, DF = 4, *P* ≤ 0.0001) and tree density (H = 22.838, DF = 4, *P* ≤ 0.0001).

**Table 1:** Environmental variables measured along the 50-m long strip plots in the lowland tropical rainforest in northeastern Leyte, Philippines.

Habitat type	Environmental variables							
	Understorey density (pole contacts) (mean ± SD)	Temperature (^o^C) (mean ± SD)	Elevation (m asl) (mean ± SD)	Leaf litter thickness (cm) (mean ± SD)	Leaf litter volume (cm^3^) (mean ± SD)	Tree DBH (cm) (mean ± SD)	Tree height (m) (mean ± SD)	Tree density (trees/ha) (mean ± SD)
PFs	3.01 ± 0.74	25.10 ± 1.40	197.88 ± 108.32	2.72 ± 0.47	2026.22 ± 641.64	15.53 ± 4.03	10.87 ± 2.11	2,675.00 ± 757.34
PFt	2.13 ± 0.58	25.86 ± 1.08	179.86 ± 101.97	3.52 ± 0.87	3,339.75 ± 803.22	16.86 ± 6.37	11.17 ± 3.00	1,371.43 ± 521.90
SLPFs	3.16 ± 0.58	26.16 ± 0.66	137.75 ± 64.59	2.35 ± 0.77	1,820.86 ± 772.75	14.69 ± 3.92	8.56 ± 1.72	1825.00 ± 1,244.13
SLPFt	2.71 ± 0.85	26.16 ± 0.46	98.29 ± 28.36	2.70 ± 0.49	2,369.90 ± 574.73	11.93 ± 2.14	8.65 ± 1.07	1685.71 ± 487.95
SFs	3.81 ± 0.81	27.37 ± 1.38	73.20 ± 24.43	1.64 ± 0.70	1776.49 ± 1,065.24	9.29 ± 1.70	6.20 ± 0.62	2,500.00 ± 827.65
SFt	4.17 ± 0.99	27.54 ± 1.25	43.67 ± 5.75	1.72 ± 0.28	1654.18 ± 594.77	7.77 ± 1.77	6.78 ± 0.99	1,483.33 ± 688.23
AFAs	0.00 ± 0.00	29.85 ± 1.86	28.00 ± 4.82	0.50 ± 0.38	399.44 ± 343.00	6.13 ± 5.33	3.40 ± 2.72	416.67 ± 416.73
AFAt	0.00 ± 0.00	30.56 ± 1.12	29.00 ± 4.74	0.30 ± 0.22	455.82 ± 429.05	2.62 ± 3.73	2.00 ± 2.83	40.00 ± 54.77
Ps	0.00 ± 0.00	29.64 ± 2.34	19.67 ± 2.08	0.19 ± 0.17	201.95 ± 231.36	5.24 ± 4.55	2.94 ± 2.57	533.33 ± 611.01
Pt	0.00 ± 0.00	29.39 ± 1.15	30.86 ± 9.79	0.23 ± 0.50	101.82 ± 213.26	1.07 ± 2.83	0.50 ± 1.32	28.57 ± 75.59

*PF* primary forest, *SLPF* selectively logged primary forest, *SF* secondary forest, *AFA* abandoned farm area, *P* pasture; *s* stream, *t* terrestrial habitats.

High correlations were found in many of the environmental variables. In stream habitat, direct high correlations were observed between elevation, leaf litter volume, leaf litter thickness and tree height (Supplementary Table S1).

### Species richness and diversity

**Figure 1:**
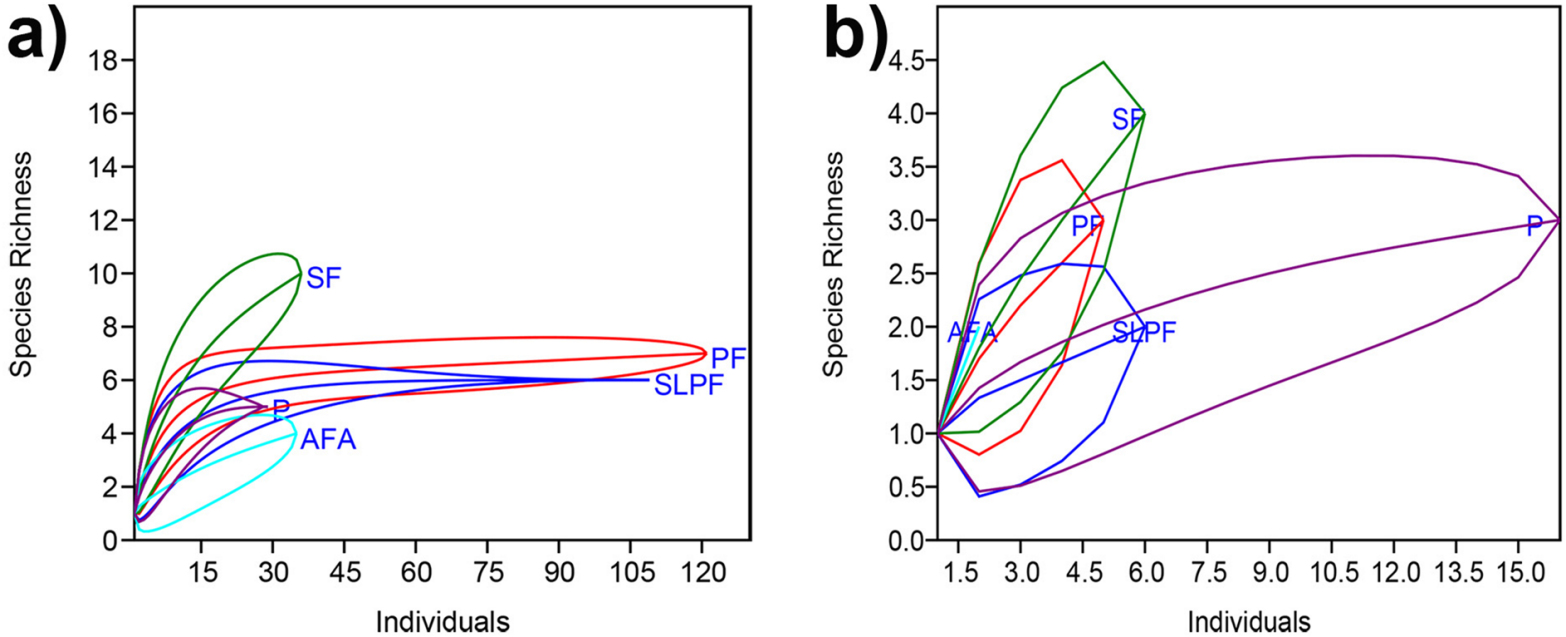
Rarefaction curves displaying sample-based species richness among habitat alteration types for (**a**) stream and (**b**) terrestrials habitats in a lowland tropical rainforest in northeastern Leyte, Philippines. *PF* primary forest, *SLPF* selectively logged primary forest, *SF* secondary forest, *AFA* abandoned farm area, *P* pasture.

In total, we observed 368 amphibians from 18 species, and belonging to 7 families (Table 2). Of all recorded individuals, most are considered as leaf-litter and semi-aquatic amphibians (360 individuals) with 15 species belonging to 6 families. Meanwhile, the remaining was considered arboreal, with 3 species belonging to the single family Rhacophoridae. Approximately one third of the total species encountered in this study are endemic to the Visayan and Mindanao Pleistocene Aggregate Island Complex (PAIC)^19^. Based on the International Union for Conservation of Nature (IUCN) red list criteria^28^; one of the amphibian species encountered was already classified as near threatened (NT), one as vulnerable (VU) and many of the remaining species were classified as least concern (LC) (Table 1). Of all the recorded amphibian species, 11% or two species (*Rhinella marina* and *Hylarana erythraea*) sampled were non-native to the country. The most common species was *Limnonetes magnus* which accounts for 22% of total individuals captured, while 50% of the species (n = 9) were represented by five individuals, and four species represented by only a single individual. The species accumulation curve for data on amphibians indicates that most species likely to occur in the stream strip plots were actually detected, except in secondary forest habitat (Fig. 1a); whereas species accumulation curve for terrestrial habitat indicated that generally, there was a likelihood of unseen species in a number of sites (Fig. 1b).

**Table 2:** The occurrence and abundance of species (all guilds) in the different habitat alteration types and habitat types in a lowland tropical rainforest in northeastern Leyte, Philippines.

Species	Code	Habitat										TS
		PF		SLPF		SF		AFA		P		
		s	t	s	t	s	t	s	t	s	t	
**Bufonidae**												
*Rhinella marina*^a^	Rm					4		1		4		LC
**Ceratobatrachidae**												
*Platymantis corrugatus*^a^	Pc		1				3					LC
*Platymantis* sp.^a^	Ps				1							
**Dicroglossidae**												
*Fejervarya vittigera*^a^	Fv							3	1	3	12	LC
*Limnonectes leytensis*^a^	Ll					1						LC
*Limnonectes magnus*^a^	Lm	16		59		5	1					NT
*Limnonectes* cf. *visayanus*^a^	Lv	11		17		14				2		VU
*Occidozyga laevis*^a^	Ol	7		13		3						LC
**Megophryidae**												
*Megophrys stejnegeri*^a^	Ms	16	3	5	5	2	1					LC
**Microhylidae**												
*Kalophrynus sinensis*^a^	Ks						1					LC
*Kaloula* sp.^a^	Ksp					1						LC
*Kaloula picta*^a^	Kp									3	1	LC
Ranidae												
*Hylarana erythraea*^a^	He							30		17	3	LC
*Pulchrana grandocula*^a^	Pg	30		5		4						LC
*Staurois* sp.^a^	Ss	40		10		1						
**Rhacophoridae**												
*Philautus leitensis*^b^	Pl	1	1									LC
*Polypedates leucomystax*^b^	Ple					1		1	1			LC
*Rhacophorus bimaculatus*^b^	Rb	3										LC

*PF* primary forest, *SLPF* selectively logged primary forest, *SF* secondary forest, *AFA* abandoned farm area, *P* pasture; *s* stream, *t* terrestrial habitats; *TS* threat status according to IUCN red list criteria (IUCN, 2019), *LC* least concern, *NT* near threatened, *VU* vulnerable.

^a^Leaf-litter or semi-aquatic species, ^b^Aarboreal species.

The GLM analyses were applied to assess the relative importance of habitat alteration types, habitat types and their interaction on the observed amphibian abundance, richness and diversity. It was found that the abundance of leaf-litter and semi-aquatic amphibians was significantly higher in forest habitats (primary forest and selectively logged primary forest) than in abandoned farm areas, and comparable in both forest and pasture habitats. In terms of habitat type, abundance was higher in stream areas versus terrestrial areas. Also, significant interaction was found between habitat alteration types and habitat types (Table 3 and Supplementary Fig. S1). Species richness varied significantly between habitat types, characterized by higher species richness in the stream habitat type (Table 3 and Supplementary Fig. S2). Lastly, diversity was significantly lower in abandoned farm areas compared to other habitat alteration types, and was significantly higher in stream areas. With respect to diversity, significant interaction was found between habitat alteration types and habitat types (Table 3 and Supplementary Fig. S3).

**Table 3:** Effect test from generalized linear model (GLM) with habitat alteration type, habitat type and interaction terms as predictor variables and abundance, richness and diversity (Shannon–Wiener) of leaf-litter and semi-aquatic amphibians as response variables.

	Abundance		Richness		Diversity (Shannon–Wiener)	
	Wald chi-square	*P* value	Wald chi-square	*P* value	Wald Chi-square	*P* value
Habitat alteration type	12.425	0.014	5.057	0.281	28.270	< 0.0001
Habitat type	93.665	< 0.0001	31.174	< 0.0001	134.319	< 0.0001
Interaction	14.352	0.006	2.135	0.711	19.119	0.001

### Species composition pattern

**Figure 2:**
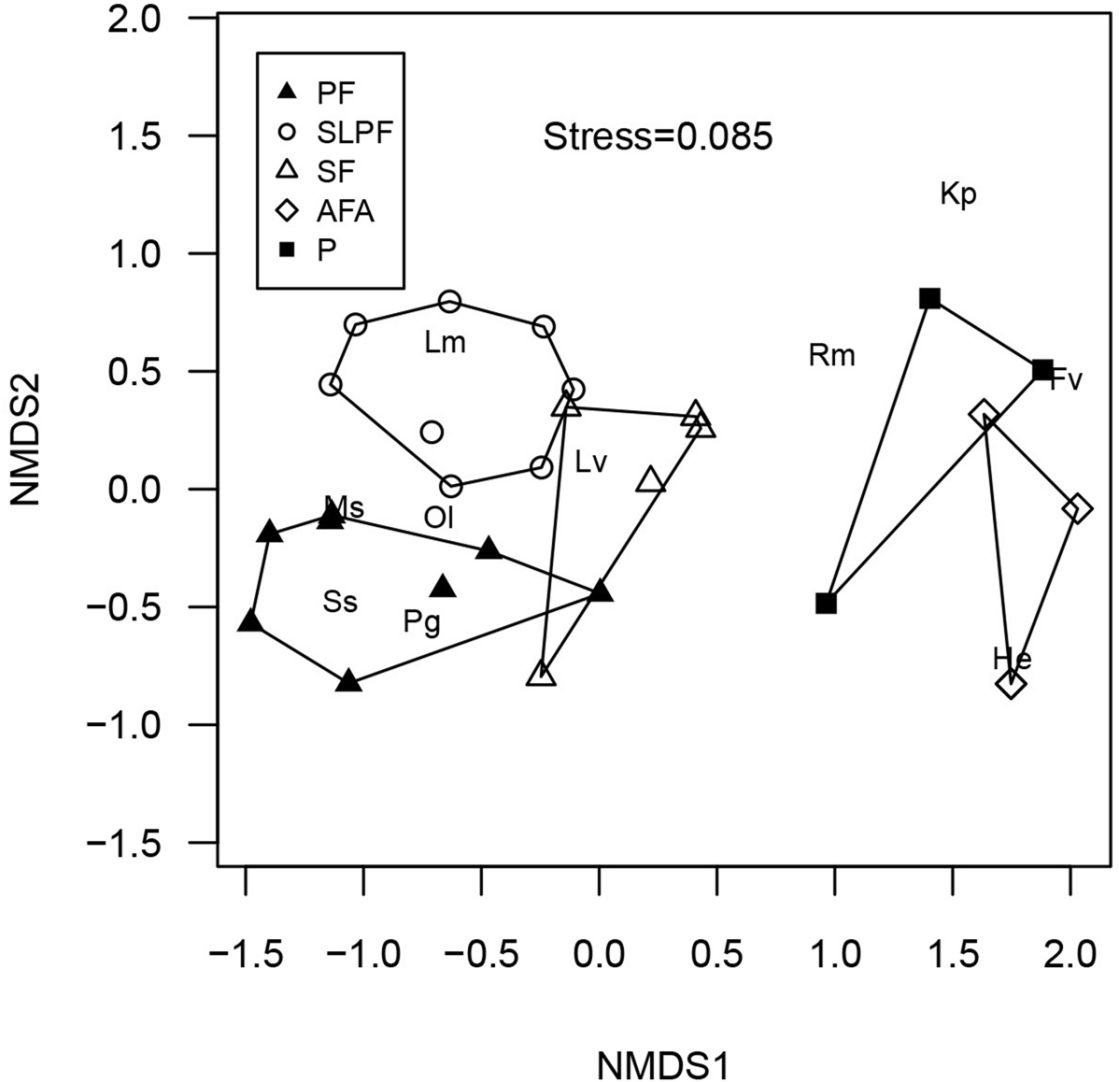
NMDS of species composition of leaf-litter and semi-aquatic amphibians among habitat alteration types for stream strip plots in a lowland tropical rainforest in northeastern Leyte, Philippines, with habitat polygons and species (two letter symbol) distribution. Solid triangle—PF; open circle—SLPF, open triangle—SF, open diamond—AFA and solid square—P. The species abbreviations are listed in Table 2.

The NMDS analysis revealed strong differentiation in leaf litter and semi-aquatic amphibian assemblage between habitat alteration types for stream strip plots, as indicated by the absence or very minimal overlap between polygons, surrounding forest and non-forest habitat alteration types in the NMDS ordination configuration (Fig. 2). This is also confirmed by the results of the ANOSIM test, wherein there was a strong and highly significant difference in assemblage composition among habitat alteration types (ANOSIM R = 0.759, *P* = 0.0002). Based on the species distribution in NMDS ordination space (Fig. 2), leaf litter and semi-aquatic amphibian species are associated with specific habitat alteration types. For example *Megophrys stejnegeri*, *Occidozyga laevis*, *Staurois* sp. and *Pulchrana grandocula* are associated with primary forest, *L. magnus* is associated with selectively logged primary forest, and *Limnonectes* cf. *visayanus* is associated with secondary forest. Meanwhile those species strongly associated with abandoned farm areas and pasture include *Kaloula picta, R*. *marina, Fejervarya vittigera* and *H. erythraea*.

### Species distribution with environmental gradients

**Figure 3:**
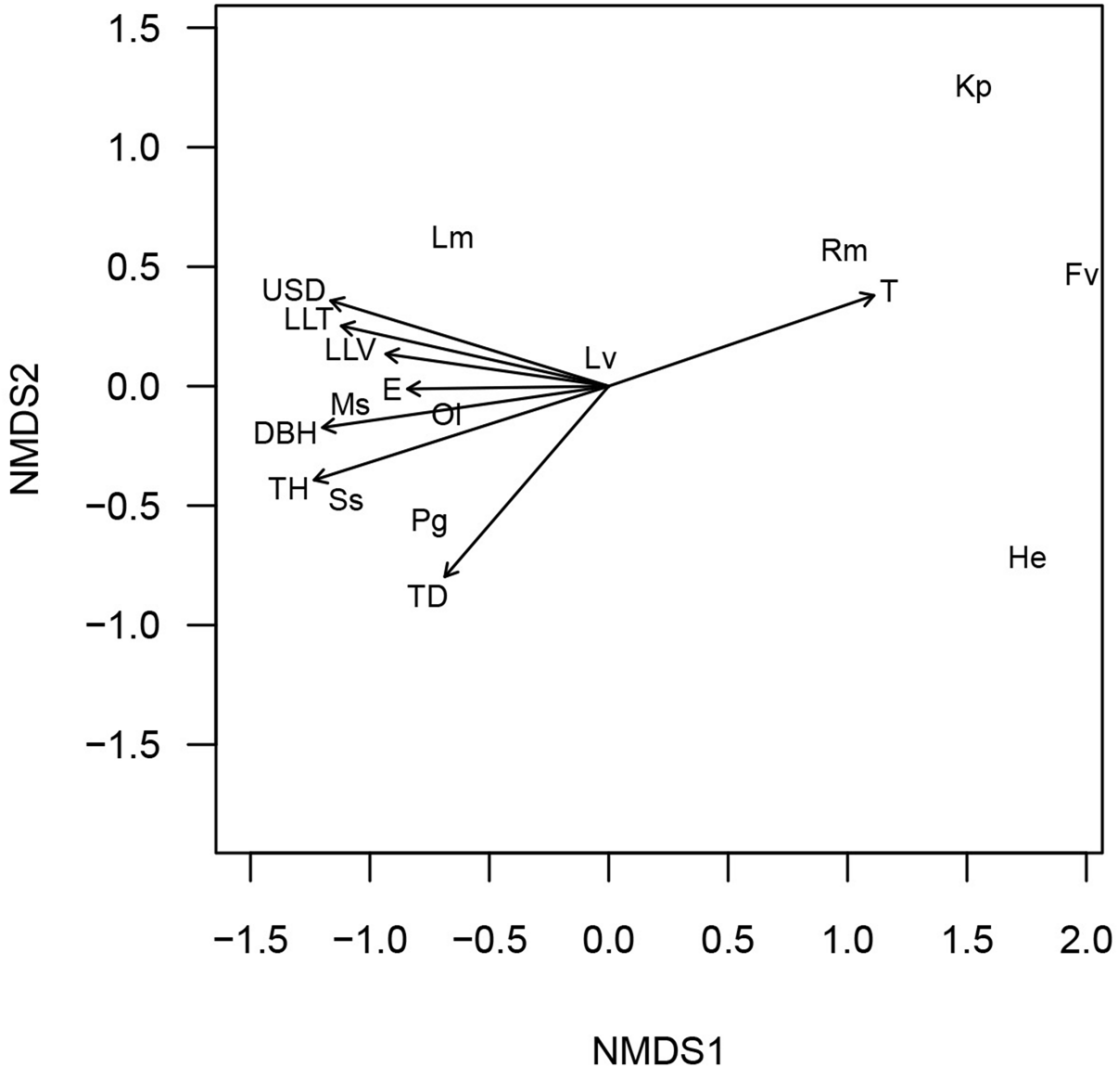
NMDS ordination showing the magnitude and direction of the fitted environmental vectors and species distribution among the different habitat alteration types for stream strip plots in a lowland tropical rainforest in northeastern Leyte, Philippines. *USD* understorey density, *T* temperature, *E* elevation, *LLT* leaf litter thickness, *LLV* leaf litter volume, *DBH* diameter at breast height (1.3 m from the ground), *TH* tree height, *TD* tree density. Two letter symbols represents species, abbreviations are listed in Table 2.

Fitting of linear vectors into ordination space indicates that all of the considered environmental variables explain variations of leaf litter and semi-aquatic amphibian assemblage in the stream habitat (Fig. 3). The permutation test indicates that all the variables significantly contribute to variations in amphibian assemblage with *P* ≤ 0.05 (Table 4). Generally, the explanatory powers of all the environmental variables are equal, and most of them are grouped together, except for temperature pointing to the right side of the ordination space. Furthermore, species distribution indicates that the forest specialist species (*L. magnus*, *M. stejnegeri*, *O. laevis*, *Staurois* sp. and *P. grandocula*) are associated with most of the environmental variables, whereas the distribution of open-habitat specialists (*K. picta, R. marina, F. vittigera* and *H. erythraea*) is determined by increasing temperature (Fig. 3).

**Table 4:** The results of fitting linear vectors to the NMDS ordination of the dissimilarity of leaf-litter and semi-aquatic amphibian assemblage.

Environmental variables	NMDS1	NMDS2	r^2^	*P*
Understorey density	− 0.956	0.294	0.588	0.001
Temperature	0.946	0.323	0.546	0.001
Leaf litter thickness	− 0.976	0.220	0.523	0.001
DBH	− 0.990	− 0.143	0.582	0.001
Tree height	− 0.953	− 0.303	0.665	0.001
Tree density	− 0.653	− 0.758	0.439	0.002
Leaf litter volume	− 0.990	0.143	0.353	0.005
Elevation	− 0.999	− 0.014	0.282	0.015

The r^2^ for each environmental vector is a measure of goodness of fit into the ordination and *P* values based on randomization test with 1,000 random permutations of environmental variables.

### Relationship between abundance, richness and diversity with environmental variables

**Figure 4:**
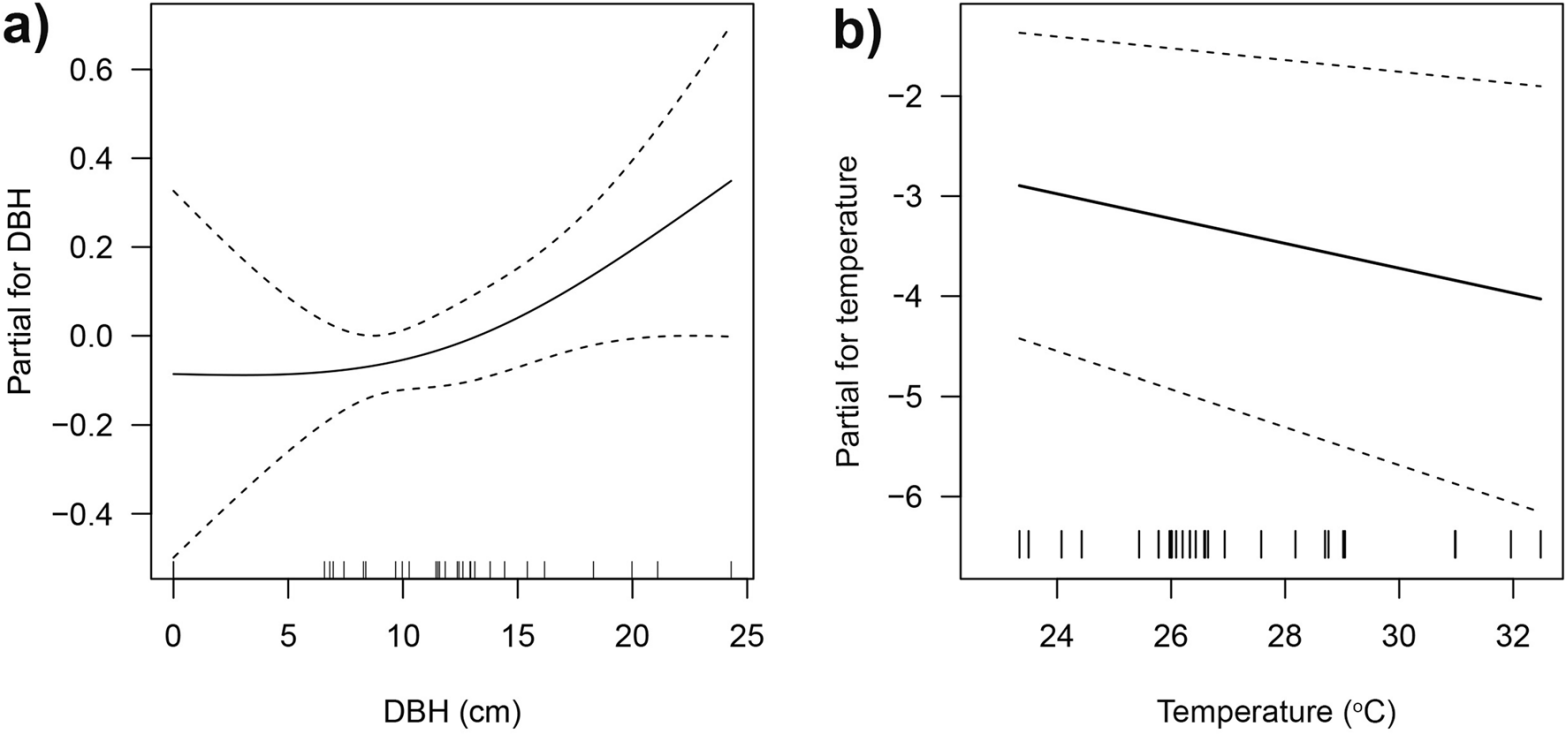
Partial effects of (**a**) DBH and (**b**) temperature in GAM model for leaf-litter and semi-aquatic amphibian abundance in a lowland tropical rainforest in northeastern Leyte, Philippines. Dashed lines indicate the standard errors for each model term.

The GAM analysis on abundance data of leaf-litter and semi-aquatic amphibians across the different habitat alteration types for stream strip plots, results in a best supported model consisting of two explanatory variables, DBH and temperature (Table 5 and Supplementary Table S2). The smoothed-term in this model shows positive non-linear relationship between abundance and DBH indicating that amphibian abundance increases with increasing DBH (Fig. 4a). On the other hand, there was a negative linear relationship between abundance and temperature indicating that abundance decreases with increasing temperature (Fig. 4b).

**Table 5:** The summary statistics for GAMs for abundance, richness and diversity relationships with selected environmental variables for leaf-litter and semi-aquatic amphibians in a lowland tropical rainforest in northeastern Leyte, Philippines.

	Estimate	SE	Z	*P*
**Abundance**				
Parametric coefficients				
Intercept	5.723	0.878	6.515	< 0.001
Temperature	− 0.124	0.033	− 3.787	< 0.001
Smooth terms	edf	df	Chi sq	*P*
s (DBH)	1.899	1.990	3.709	0.137
Adjusted R^2^	0.242			
**Richness**				
Parametric coefficients				
Intercept	1.170	0.104	11.240	< 0.001
Smooth terms	edf	df	Chi sq	*P*
s (Understorey density)	1.794	1.958	8.224	0.027
Adjusted R^2^	0.293			

	Estimate	SE	t	*P*
**Diversity**				
Parametric coefficients				
Intercept	0.958	0.075	12.740	< 0.001
Smooth terms	edf	df	F	*P*
s (Understorey density)	1.403	1.640	2.137	0.168
s (Tree density)	1.331	1.551	2.649	0.102
s (Temperature)	1.410	1.650	1.414	0.357
Adjusted R^2^	0.359			

GAM for abundance includes linear term for temperature and smoothed-term for DBH; GAM for species richness only includes smoothed-term for understorey density; GAM for diversity (Shannon–Wiener) includes smoothed-terms for understorey density, tree density and temperature.

**Figure 5:**
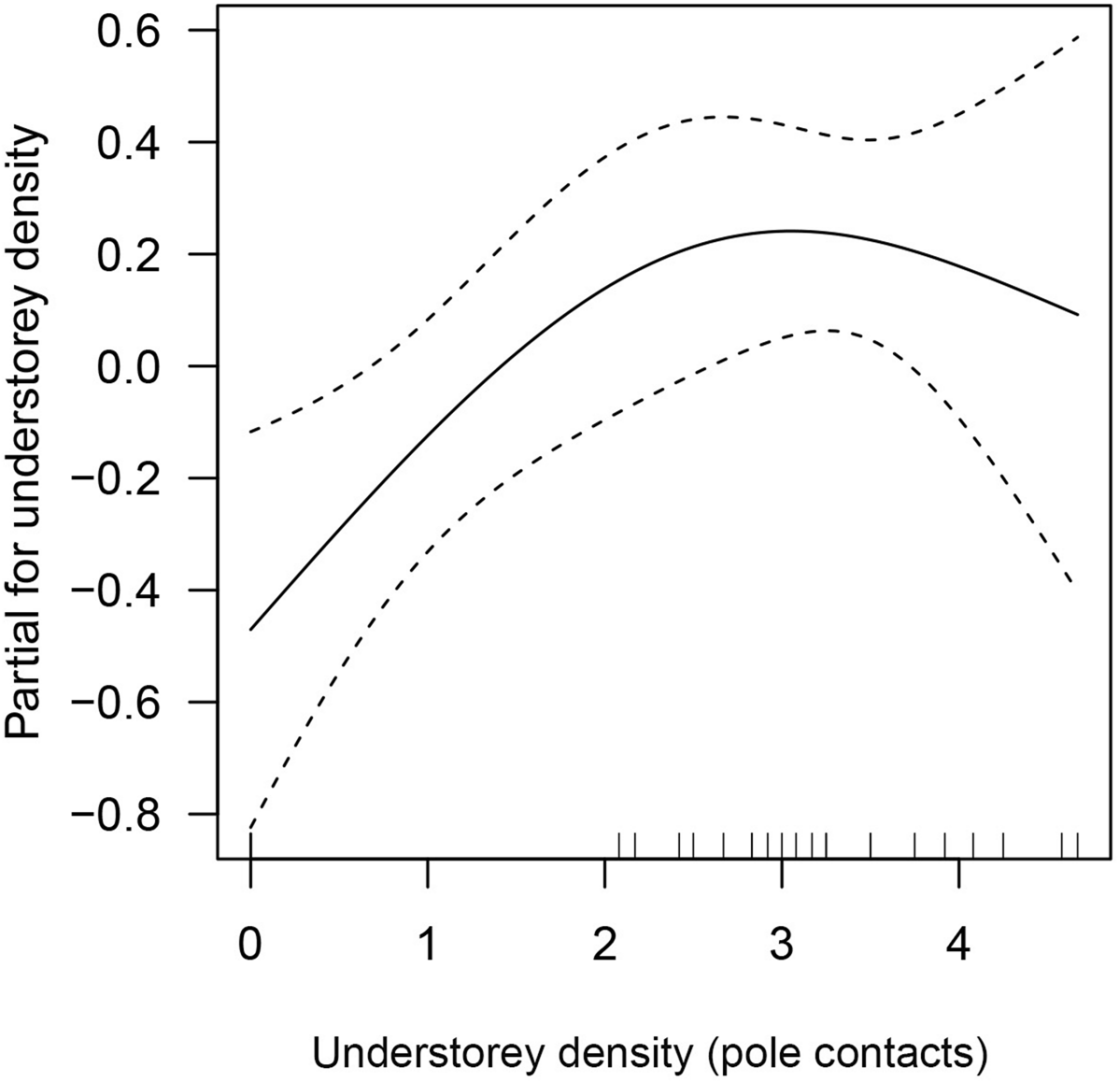
Partial effects of understorey density in GAM model for leaf-litter and semi-aquatic amphibian richness in a lowland tropical rainforest in northeastern Leyte, Philippines. Dashed lines indicate the standard errors for the model term.

The best supported model for leaf-litter and semi-aquatic amphibian richness, in the different habitat alteration types for stream strip plots, includes only understorey density (Table 5 and Supplementary Table S2). The model shows non-linear relationship between amphibian richness and understorey density, where amphibian richness peaked at higher understorey density (Fig. 5).

**Figure 6:**
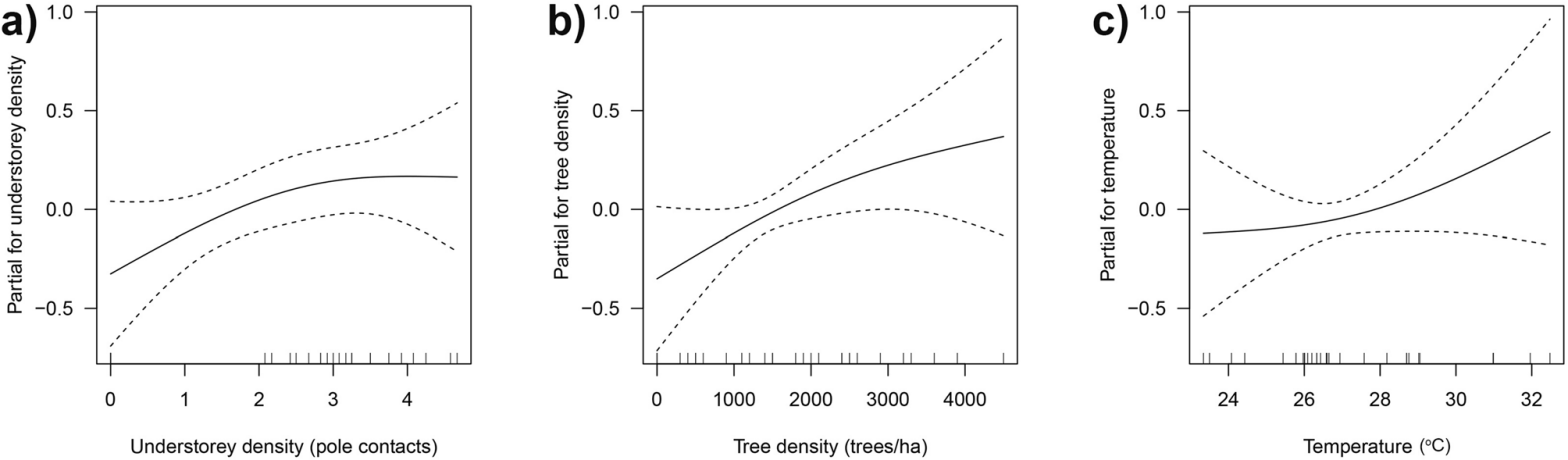
Partial effects of (**a**) understorey density, (**b**) tree density, and (**c**) temperature in GAM model for leaf-litter and semi-aquatic amphibian diversity (Shannon–Wiener) in a lowland tropical rainforest in northeastern Leyte, Philippines. Dashed lines indicate the standard errors for each model term.

Lastly, the best supported model for leaf-litter and semi-aquatic amphibian diversity includes three explanatory variables, including understorey density, tree density, and temperature (Table 5 and Supplementary Table S2). All three smoothed-terms show positive non-linear relationships, wherein diversity increases with increasing understorey density, tree density and temperature (Fig. 6).

## Discussion

Based on the findings, habitat alteration influences abundance and diversity but not species richness of leaf-litter and semi-aquatic amphibians in the lowland tropical rainforest in northeastern Leyte. In addition, in the study area, abundance, richness and diversity were highly dependent on habitat type (stream and terrestrial). In general, the abundance and diversity of leaf-litter and semi-aquatic amphibians was significantly higher in less disturbed habitat (e.g., primary forest and selectively logged primary forest) compared to the severely altered habitat (e.g., abandoned farm areas and pasture). A reduction in species abundance and diversity in the altered habitats may well be attributed to the decrease in habitat structural complexity through habitat degradation^29^. For example, the loss of primary vegetation would be detrimental to some amphibian species especially to those forest specialists or leaf-litter species. Likewise, the loss of habitat components can lead to changes in microclimatic conditions such as temperature, some of which can be unfavorable to amphibians, due to increased risk of dehydration^8^. Less disturbed primary forest was mainly dominated by forest specialist species such as *Staurois* sp*.*, *P. grandocula* and *M. stejnegeri* (which had seen notable reductions or were altogether absent from the altered habitats). The lack of difference in species richness between habitat alteration types, for leaf-litter and semi-aquatic amphibians, shows that forest alteration and/or land use conversion in the study system may not significantly impact the number of species^9,30,31^. Additionally, this may help explain why many of the leaf-litter and semi-aquatic amphibian species in primary forests are also observed in the selectively logged primary forests and secondary forests. On the other hand, as a result of habitat degradation, the disappearance of forest specialist species in highly altered habitats (e.g. abandoned farm areas and pastures) likely leads to replacement by open-habitat specialists (e.g. *H. erythraea* and *F. vittigera*). Although species richness did not differ between habitat alteration types, species composition changed markedly (see NMDS ordination plot and ANOSIM). While this study did confirm that highly modified habitats (e.g. plantations, abandoned farm areas and pastures) do host a number of amphibian species^32,33^, primary forests and secondary forests should remain a higher priority target for conservation effort, since these habitat host greater number of forest specialist species^30^ such as *Staurois* sp*.*, *P. grandocula* and *M. stejnegeri*. The higher amphibian abundance, richness and diversity in the stream, as compared to terrestrial habitat, indicated that amphibians in the study area exhibited proximal dependence on water sources and areas which likely provide them with crucial breeding habitat. In fact, species including *L.* cf. *visayanus*, *O. laevis*, *P. grandocula*, *Staurois* sp. and *R. marina* were exclusively observed in the stream habitat and tadpoles of some of these species were observed in waters along stream banks and small isolated pools of water. Also, some species use both stream and terrestrial habitat to complete their life cycle (e.g. *M. stejnegeri*)^28^. Higher recorded abundance and diversity in the streams is likely related to the mountainous terrains, with almost no other water resources such as the ponds vital for reproduction^9^. On the other hand, human activity, particularly hauling of logs in both forested and non-forested areas, may contribute to the creation of puddles that serve as an ephemeral breeding habitat for amphibians during the rainy season (i.e. Microhylidae). Based on the observation and analysis, it follows that a greater number of stream-dependent species may be adversely affected if there is continued alteration to environmental components of the stream ecosystem. For example, drier conditions associated with significant alteration, potentially leading to reduced streamflow, and additionally, increased rate of precipitation runoff resulting in stronger torrents, are both factors that would likely result in higher amphibian mortality, and decline in the amphibian population^34^.

One of the key findings is that there are few individuals and few species of amphibians observed in terrestrial strip plots (in many cases, researchers did not observe a single individual). The recorded abundance and diversity in terrestrial habitat was contrary to the results of a number of noted studies conducted in tropical rainforests^9,32,35^. It is important to note that low rates of observation in the study area may have reflected low densities or low detection probability^36^. The preceding effect is likely influenced by the limitations of the sampling method, where visual encounter survey was the sole method employed. In a few plots, arboreal species *P. leitensis* were suspected to call from arboreal habitats such as aerial ferns, tree branches and understorey vegetation. It is important to note, particularly for future effort, that difficult-to-sample species with arboreal characteristics could be efficiently accounted for using an auditory survey method. However, based on observations, we suspect the terrestrial habitats in the study area were not likely rich in arboreal species, which includes *P. leitensis* and the rarely encountered *Theloderma spinosum*. The genus *Philautus* are one of the dominant groups of amphibians that inhabit the terrestrial habitat, but most of these species were documented in the Mindanao PAIC, and typically inhabit primary forests at higher elevations^19,23,26^. As a matter of sampling concern, increased sampling of terrestrial strip plots with respect to stream strip plots, could still prove insufficient for overall sampling methodology. As a recommendation in disturbed forest habitat, amphibians could potentially be sampled using the acoustic method, particularly those species calling in hidden places such as thick bushes/grasses^35^ and river undercuts.

The assemblage of leaf-litter and semi-aquatic amphibians in stream strip plots differed markedly between each habitat alteration type, and also between forests and non-forest habitat. Primary forest was strongly associated with the following forest specialist species: *M. stejnegeri*, *O. laevis*, *Staurois* sp. and *P. grandocula*. In terms of reproduction, these species were predominantly stream dependent within primary forests, wherever there was evidence of breeding found in streams. To illustrate, the eggs and tadpoles of the common paddle frog *O. laevis* were almost exclusively found in the small isolated pools of water along the riparian zones in the study area, although the species is also known to breed in marshes^28^. For the disturbed forest habitat, amphibians under family Dicroglossidae (*L*. *magnus* and *L*. cf. *visayanus*) were most commonly found. These species are leaf-litter or forest specialists and simultaneously stream dependent, as they breed in aquatic environments to complete their life cycle. In fact, eggs of *L*. cf. *visayanus* were observed on rock surfaces along stream banks, where, once eggs hatch, tadpoles likely move to the stream water where they develop^28^. By contrast, the amphibian assemblage for non-forest habitat (abandoned farm areas and pasture) is dominated by semi-aquatic and open-habitat specialist species. These habitat types were highly dominated by the widespread alien species *H. erythraea*, and *F. vittigera,* which has been established as an endemic Philippine species inhabiting the low elevation aquatic habitats and is often found in drainage ditches and rice fields^19^.

Among habitat alteration types the species composition of leaf-litter and semi-aquatic amphibians for stream strip plots varies significantly along the gradient of habitat structure and microclimatic condition (Fig. 3). Our results indicate that changes in habitat structure and microclimatic condition, as related to implied habitat alteration, are accompanied by changes in species composition^37^. Forest habitat alteration type is more structurally diverse compared to abandoned farm and pasture area, and thus, the majority of habitat variables explained species composition in riparian areas of forest habitat alteration types. For example, understorey density provides good habitat quality for forest species^14^ which may also provide understorey cover for leaf-litter species. Thick leaf litter may help maintain microhabitat quality, resulting in favorable conditions such as moisture content which are particularly beneficial for amphibian species^37^. In previous studies, vegetation structures (i.e. DBH) also appeared to be an important factor in the occurrence of amphibian species^36,37^. Generally, the larger tree diameters found in less disturbed primary forest are associated with greater amphibian richness. Conversely, changes in microclimatic condition, such as increase in ambient temperature, has been implicated in loss of certain structural aspects of the habitat^29^. Within the study area, abandoned farm areas and pasture areas are characterized by elevated temperature, and accordingly, may have favored amphibian assemblages advantageous to open-habitat specialist species. Although open environments are typically hostile to many of the ectothermic amphibians^8^, the existence of some species in an open environment in the study area could be indicative of the use of other forms of microhabitats which were absent in forest habitats. To illustrate, *H. erythraea* was commonly observed in areas extensively covered with thick matt-forming perennial herb *Sphagneticola trilobata*, so the species may have the ability to conceal itself inside the thick matt under intense sunlight. Additionally, other species may be using undercuts in banks as shelter in stream areas with few or no trees.

The abundance of leaf-litter and semi-aquatic amphibians showed relationships to DBH and temperature. Amphibian abundance increased with increasing tree DBH, confirming this habitat structure as an important factor in determining the presence of amphibians^14,37^. Based on our observations, forest habitats with large trees, particularly primary forests, served as the primary habitat for leaf-litter species such as *M. stejnegeri* and *Limnonectes* spp. Alternatively, amphibian abundance decreased with increasing air temperature. Notably, stream strip plots for abandoned farm areas and pasture habitats are characterized by higher temperature and lower abundance of leaf-litter and semi-aquatic amphibians. Under elevated temperature, dissolved oxygen in water is reduced resulting in decline in swimming and invasion performance of tadpoles, leading to increased mortality^34^. While it is generally understood that amphibians are negatively affected by elevated temperature, the observed increase in temperature in the study area is primarily associated with the loss of structural aspects of habitat (e.g. canopy cover and understorey density) due to disturbance^29^. Similarly, this change in microclimatic condition may result in desiccation of spawn mass, particularly for species spawning in leaf litter^29^. This effect could explain the lower number of individuals or absence of *L.* cf. *visayanus* in more altered habitat (abandoned farm areas and pastures) since eggs are deposited outside the water, typically on vegetation or rocks just above the water^28^. In interior forest areas, *Platymantis corrugatus* could potentially be affected by the increased temperature since the species breeds and lays its eggs in leaf-litter, and breeds by direct development^28^.

Habitat heterogeneity largely influences the richness and diversity of amphibians^36,38^. Whilst, in this study, species richness was related to understorey density only, previous studies have determined that patterns of amphibian richness can be influenced by wide environmental gradients^14,33,36,39^. In the study area, species richness of leaf-litter and semi-aquatic amphibians peaks in conjunction with higher understorey density. In the riparian ecosystems, lower understorey density is an important structural component of the habitat since this serves as calling and resting sites for arboreal species^38^ and also provides vital understory canopy cover for those leaf-litter species. Lastly, diversity of leaf-litter and semi-aquatic amphibians is positively associated with environmental variables/habitat structures such as understorey density, tree density and temperature. The proportional relationship between diversity and tree density observed in this study was likewise observed in existing literature^40^. Higher tree density provides the necessary habitat conditions for typical rainforest composition of amphibian species^41^. This could be associated with greater habitat heterogeneity (e.g. increase canopy cover and thick leaf-litter cover) and microclimatic amelioration (e.g. lower temperature), as both conditions offer better habitat quality. Increased tree density through forest restoration may facilitate the recolonization of specialist species^42^, and thus, lower tree density in the degraded habitats (abandoned farm areas and pasture areas) likely accounts for the absence or reduced number of amphibians (forest specialists). Moreover, temperature had an increasing effect on the diversity of amphibians which was in contrast to the observed effect of temperature on abundance. This could be potentially explained by the high number of species and stable population of open-habitat specialist species in abandoned farm areas and pasture (e.g. *H. erythraea*, and *F. vittigera*, *R. marina*), though such trends could differ if all guilds of amphibians be taken into account.

The results of the study indicate that habitat alteration mainly affected the diversity and species composition of leaf-litter and semi-aquatic amphibians. Of the habitats examined, the stream habitat had the greatest abundance, species richness and diversity, thus highlighting a potential higher priority target for conservation. In addition, habitat alteration is attributed to reduction in habitat structural complexity, and changes in microclimatic condition, which in turn influence the species composition, abundance, richness and diversity. Within the study area, less disturbed habitat areas are important for the maintenance of amphibians, particularly the leaf-litter and semi-aquatic species, and thus, merit continued evaluation and prioritization for conservation efforts (this encompasses stream habitats with higher understorey density, lower temperature, larger trees and higher tree density). Lastly, the diversity of the concerned species are undeniably threatened by continuous habitat loss (e.g. mainly “Kaingin” or slash-and-burn agriculture and illegal logging) and thus there is impetus to develop and implement effective conservation and management plans.

## Methods

### Study area

**Figure 7:**
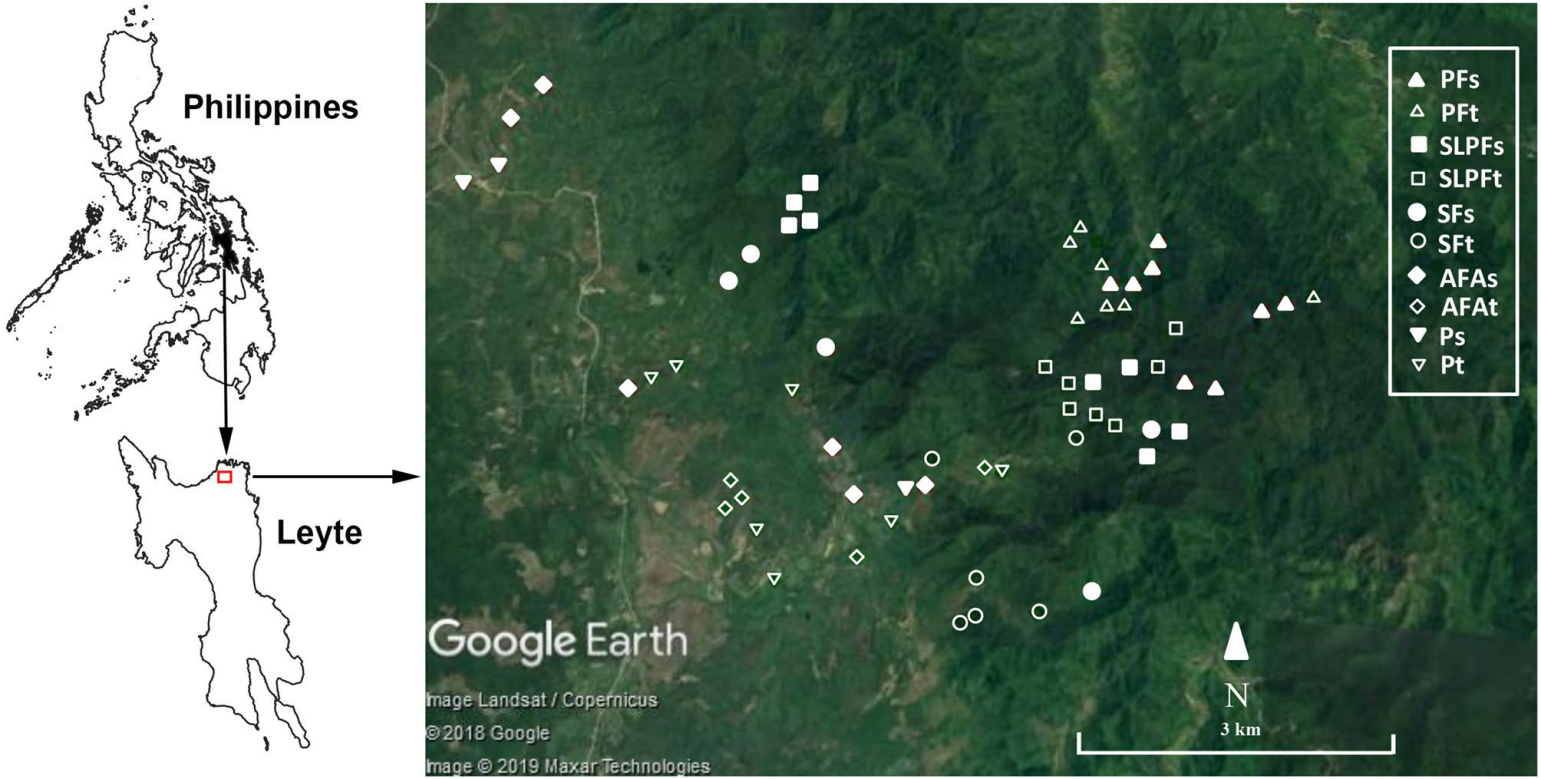
Map of the area and study plots in a lowland tropical rainforest in northeastern Leyte, Philippines. *PF* primary forest, *SLPF* selectively logged primary forest, *SF* secondary forest, *AFA* abandoned farm area, *P* pasture; *s* stream, *t* terrestrial habitats.

The study was conducted in a tropical rainforest in Babatngon Range in the northeastern portion of Leyte Island (Fig. 7) which covers two municipalities- San Miguel and Babatngon. The core of the mountain range is represented by ultramafic outcrops known as the Tacloban Ophiolite Complex (TOC). It is a NW–SE trending massif in the northeastern portion of the island, overlain by sedimentary sequences dated to Late Miocene-Pliocene and Pleistocene volcaniclastic deposits on its eastern and western flanks, respectively^43^. The maximum elevation of the study area reaches up to 600 m a.s.l with an average elevation range from 19.67 to 197.88 m a.s.l and with a median and interquartile range (iqr) of 68.50 (102.00 m a.s.l.). Under the Modified Corona’s Climate Classification System, the study area has a Type IV climate characterized by having no dry season and more or less evenly distributed rainfall throughout the year^44,45^. The warmest month is April with an average temperature of 28.1 °C and the pronounce wetness occurs in the months of November, December, and January with rainfall of 279.0 mm, 305.3 mm, and 281.17 mm, respectively^46^. Like in many tropical forest ecosystems, the predominant causes of forest degradation in the hilly portions of the study area were the slash-and-burn agriculture (“Kaingin”) which involves clearing and burning of forests for agricultural purposes, and illegal logging. Whereas the lowlands are already converted to rice paddies which are mostly rain fed. These resulted in the degradation and alterations of forest ecosystems in the study area.

### Sampling sites

Prior to the sampling, ground surveys were conducted by visiting the study area to identify the habitats for amphibian sampling, these included primary forest (less altered), selectively logged primary forest, secondary forest, abandoned farm areas and pastures (highly altered), representing a gradient of habitat alteration. In addition, informal interviews with the local residents and owners were conducted to know the land use histories of the study sites selected. All the forest types included in this study were all contiguous.

#### Primary forest

The primary forests are considered to be less disturbed having no significant human disturbance primarily logging. Primary forests tend to be located in protected watershed areas and in areas with very steep terrain. These primary forests are characterized by unlogged and intact dipterocarp forests with canopy reaching 30–50 m high. The intent to sample pristine and undisturbed forest was complicated by the fact that much of the primary forests in the study area have already been subjected to minor disturbance such as rattan harvesting and wildlife hunting and collection (e.g. wild pig, birds and orchids).

#### Selectively logged primary forest

The selectively logged primary forests were characterized by traces of old and newly cut trees, as well discarded woods were commonly observed along the streams. These logged forests had very few or no remaining dipterocarps although some other large trees could still be observed. In addition, these forest areas were also frequently subjected to rattan harvesting and wildlife hunting.

#### Secondary forest

The secondary forests varied from 5 to 15 years old. All secondary forest stands have similar past land use, having been cleared for agricultural purposes, and have regenerated since abandonment. The vegetation composition of the secondary forests was predominantly characterized by early successional and fast growing trees species (e.g. *Commersonia bartramia*, *Ficus* spp. and *Piper aduncum*) and large long-lived pioneer species (e.g. *Artocarpus* sp. and *Cananga odorata*). However, some remnants of agricultural crops (e.g. banana and coconut) were still observed in these areas.

#### Abandoned farm area

The abandoned farm areas were characterized mainly by grasses with bushes like melastoma (*Melastoma* sp.) and guava plant (*Psidium guajava*). Although few tree species might be present including the exotic Gmelina tree (*Gmelina arborea*). Few of the abandoned farm areas were very seldom subjected to grazing.

#### Pasture

The pasture areas sampled in this study are permanently grazed, and are usually adjacent to abandoned farm areas or forest remnants. The pasture areas are typically either abandoned paddy fields or subsistence crops. These areas are marked by the sparse presence of trees or bushes, with a thin layer of grasses.

### Amphibian sampling

To sample amphibian species, a total of 62 unique 2 m × 50 m strip plots^6,9,35^ were laid with 15 strip plots in the primary forest (8 stream and 7 terrestrial strip plots), 15 in selectively logged primary forest (8 stream and 7 terrestrial strip plots), 11 in secondary forest (5 stream and 6 terrestrial strip plots), 11 in abandoned farm areas (6 stream and 5 terrestrial strip plots) and 10 in pastures areas (3 stream and 7 terrestrial strip plots). To sample stream breeding amphibians, 1 m wide riparian vegetation in both banks of the stream including the water body were searched. As well, a 2 m wide strip plots in the terrestrial (interior) were laid 50 m away from the nearest stream whenever possible^9^. Similarly, stream strip plot of the same dimension were laid in non-forest habitats (abandoned farm areas and pasture) and we made sure that the section of the stream had the same habitat type for both banks. The terrestrial strip plots were usually close and parallel to trail, in case of abandoned farm areas. The strip plots were generally straight linear however, deviated wherever there were obstacles, or paths were constrained by topography, and stream pattern. The strip plots were placed away from habitat edges as much as possible, though sometimes they could not be avoided in small forest areas (particularly in the young secondary forests). The distance between the strip plots was kept to a minimum of 300 m in forest sites and 200 m in non-forest sites. To maximize sampling independence, only one strip plot in each patch of non-forest habitat (abandoned farm areas and pasture) was established, and each separated by stream or forested area. Also, to minimize the seasonal confounding effects, different combinations of strip plots from the different habitat types were sampled throughout the sampling period^36^ whenever possible. All the strip plots were positioned using a hand-held GPS (Garmin etrex).

The amphibian sampling took place from November 2017 to December 2018. This study used only visual encounter survey^9,31,41^ whereby each strip plot was thoroughly searched for amphibians twice in the same day, first in the morning at 0800 to 1100 h, and second in the night at 1900 and 2200 h (in order to sample both diurnal and nocturnal species). Since only visual encounter survey was employed, only leaf-litter and semi-aquatic amphibians were effectively sampled, though all guilds of amphibians encountered were recorded. All the strip plots received a second session of day and night sampling in the same day, in at least one week, thus every strip plot received a total of four sampling sessions^9^. In this study, a total of 248 sampling sessions were conducted. Two people (with head torch for night sampling) slowly progressed at ground level and thoroughly searched and recorded every amphibian species that were encountered and captured. This included searching numerous kinds of substrates or surfaces such as leaf litter, rocks, soil, fallen or rotten logs, shrubs, tree trunks, branches and leaves^27^. Amphibian surveying was limited vertically to 3 m above ground level, which generally represents a restriction in the surveyors’ ability to detect individuals. The speed of the sampling per plot was approximately 3 m/min except during handling and recording. A total of two to three strip plots were sampled for each day and night survey depending on the topography and distance between strip plots except for one occasion where only one strip plot was sampled. To minimize the disturbance in the study system, logs were not displaced and plants were not cut or uprooted except when there were vines obstructing the transect. All individuals captured were marked via toe clipping^47^ and measured for snout-vent length (SVL) in mm. The individuals sampled were released unharmed at the point of collection after taking measurements and photographs.

Amphibians sampled in the field were identified and photographed for vouchering. Amphibian species were identified following existing relevant literature on Philippine amphibians^18,19,24^ particularly for the Mindanao PAIC and as well AmphibiaWeb^48^ and International Union for the Conservation of Nature (IUCN) red list criteria^28^ were consulted for further verification.

### Environmental variables

Environmental variables such as elevation, DBH, tree height, tree density, air temperature, understory density, leaf litter thickness, and leaf litter volume were measured to determine whether they affect the pattern of amphibian assemblage particularly leaf-litter and semi-aquatic species in the different habitat alteration types and habitat types. The elevation of each strip plot was recorded from a handheld GPS reading (accuracy: ± 3 m). All the trees with a DBH of ≥ 5 cm along the strip plots with a total area of 100 m^2^ were sampled. The total number of trees was counted as well as DBH was measured and the tree height was visually determined through the use of a 2-m long calibrated pole^49^. The values for DBH and tree height were averaged for each strip plot. Air temperature at each strip plot was measured using a thermohygrometer (accuracy: ± 1 °C) after 10 s of exposure for the two day sampling period. The understorey density was measured by placing a 2-m-long pole with a diameter of 2 cm perpendicular to the ground at the center of the strip plot in the interior forest, and the number of leaves touching the pole was counted^9^. The thickness of leaf litter was determined by inserting a sharpened wooden dowel (3 mm diameter) into the litter. The top portion of the litter was marked along the wooden dowel and the surrounding leaf litters were removed aside until reaching the humus layer of the soil. Afterwards, the difference between the soil layer and the marked portion of the wooden dowel was measured using a graduated ruler^50^. Determining leaf litter volume was carried out by collecting leaf-litter from an area of 1 m^2^ and pressing the leaf litter samples in a bucket of known volume (5,000 cm^3^) and measuring the height (cm) of the column^51^. The measurements for air temperature, understorey density, leaf litter thickness and leaf litter volume were done at an interval of 10 m along the strip plot and the values were averaged also. The environmental parameters (understorey density, leaf litter thickness and volume) were measured for both banks for stream strip plots.

### Data analysis

The abundance (total number of individuals encountered/recorded), species richness and diversity of leaf-litter and semi-aquatic amphibians were determined for each strip plot across habitat alteration types (primary forest, selectively logged primary forest, secondary forest, abandoned farm areas and pasture) and for every habitat type (stream and terrestrial). Individual rarefaction curves were generated to illustrate the pattern of species richness and adequacy of sample size among habitat alteration types for stream and terrestrial strip plots. Diversity index and rarefaction curves were calculated using the PAST 3.22^52^. Presence of autocorrelation in spatial data is a common issue that indicates dependence between observations^53^, hence, spatial autocorrelation analysis (Moran’s I) was performed in *R* package *ape*^54^. The Moran’s I test was performed for the data on abundance, species richness and diversity by using the mid-point geographical coordinates of each strip plot. Though Moran’s I test for all the variables were significant, the Moran’s I values were very close to 0 (Supplementary Table S3) suggesting very minimal spatial autocorrelation^55^.

Spearman’s correlation was performed to determine correlated and non-correlated environmental variables. To study the differences in measured environmental variables and to confirm the a priori classification of habitat disturbance, Kruskal–Wallis ANOVA was performed for both stream and terrestrial habitats separately. Generalized linear models (GLMs) were carried out to assess the effects of habitat alteration types (primary forest to pasture) and habitat types (stream and terrestrial) including their interactions to amphibian abundance, richness and diversity. The GLMs analysis used poisson distribution with logarithmic link function for count data (abundance and richness) whereas normal distribution with identity link function was used for continuous data (diversity). In addition, the ratio between the degrees of freedom (df) and deviance of all the models were below 2.5 indicating acceptable model fit. Both the Kruskal–Wallis ANOVA and GLMs analysis were performed using SPSS 20 for Windows.

The non-metric multidimensional scaling (NMDS) ordination was applied to explore the variation of amphibian assemblage across the different habitats^14,30,35^. For this study, NMDS was performed whereby the ordination was constructed from the Bray–Curtis dissimilarity matrix of pairwise dissimilarities between transects based on abundance data. The NMDS was performed using the function “metaMDS” from *R* package *vegan*^56^. In constructing the ordination diagram, twenty random starting configurations were used, with the final configuration that minimized the stress of the ordination configuration retained for plotting. The NMDS ordination projects the multivariate data into a space with a smaller number of dimensions. They are arranged in space in such a way that the most similar sites are close together, while sites that are more different are further apart^36^. The Analysis of Similarities (ANOSIM) permutation tests in *vegan* package of *R*^56^, with 5,000 random permutations of the dissimilarity matrix was performed to test the differences in species assemblage of leaf-litter and semi-aquatic amphibians across habitat alteration types for stream strip plots.

To explore the relationship between the distribution of leaf-litter and semi-aquatic amphibian species and environmental variables in a gradient of habitat alteration, linear vectors were fitted into the NMDS ordination using the function *envfit* in the *vegan* package of *R*^56^. This was done by running first an NMDS ordination of species and then followed by fitting the linear vectors (environmental variables) simultaneously. The analysis used the same species abundance data, and environmental variables fitted into the ordination included elevation, tree density, tree height, DBH, temperature, leaf litter thickness, leaf litter volume and understory density. The significance of the environmental vectors was evaluated using a permutation test with 1,000 random permutations.

Lastly, the relationship between the abundance, species richness and diversity of leaf-litter and semi-aquatic amphibians with environmental variables were examined using the Generalized Additive Model (GAM) in *R* package *mgcv*^57^. The GAM performed for abundance and species richness (count data) used Poisson error structure and logarithmic link functions while Gaussian error structure and identity link function for diversity (continuous data). All the environmental variables with correlations > 0.65 were excluded in the analysis, thus, only understorey density, tree density, temperature and DBH were considered.

In performing the GAM, a full model was first fitted with smooth-terms for all the selected environmental variables. With the initial fitting, some of the environmental variables were best fitted by smoothed-terms with effective degrees of freedom (edf) equal to one indicating simple linear relationships. Thus, in the succeeding fitting, these terms were expressed into linear terms. The final model was selected by dropping the least significant environmental variables one at a time until the Akaike’s Information Criterion (AIC) no longer improved. In addition, residual plot for each of the model was also checked. The shape of the response curves associated with each term was illustrated by plotting the partial effects.

The NMDS, ANOSIM, fitting of linear vectors and GAM analyses were only performed for habitat alteration types for the stream strip plots with the use of *R* software version 3.4.3^58^. This was due to insufficient samples (species counts or abundance) for terrestrial habitat type. In addition, due to limitation in sampling methodology used wherein visual encounter survey was only performed, some arboreal species were probably missed. Thus, we excluded arboreal species in all the statistical analyses and focused only on leaf-litter and semi-aquatic species. Also, singletons were excluded in all the analyses.

## Supplementary information

Supplementary file1 (PDF 513 kb)

## References

[1] Langenberger G, Martin K, Sauerborn J (2006). Vascular plant species inventory of a Philippine lowland rain forest and its conservation value. Biodivers. Conserv..

[2] Sodhi NS, Koh LP, Brook BW, Ng PKL (2004). Southeast Asian biodiversity: an impending disaster. Trends Ecol. Evol..

[3] Lasco RD, Veridiano RKA, Habito M, Pulhin FB (2013). Reducing emissions from deforestation and forest degradation plus (REDD+) in the Philippines: will it make a difference in financing forest development?. Mitig. Adapt. Strateg. Glob. Change.

[4] Liu DS, Iverson LR, Brown S (1993). Rates and patterns of deforestation in the Philippines: application of geographic information system analysis. Forest Ecol. Manag..

[5] Sodhi NS (2010). Conserving Southeast Asian forest biodiversity in human-modified landscapes. Biol. Conserv..

[6] Riemann JC, Ndriantsoa SH, Rödel M-O, Glos J (2017). Functional diversity in a fragmented landscape–habitat alterations affect functional trait composition of frog assemblages in Madagascar. Glob. Ecol. Conserv..

[7] Peltzer PM, Lajmanovich RC, Attademo AM, Beltzer AH (2006). Diversity of anurans across agricultural ponds in Argentina. Biodivers. Conserv..

[8] Cruz-Elizalde R, Berriozabal-Islas C, Hernández-Salinas U, Martínez-Morales MA, Ramírez-Bautista A (2016). Amphibian species richness and diversity in a modified tropical environment of central Mexico. Trop. Ecol..

[9] Jongsma GFM, Hedley RW, Durães R, Karubian J (2014). Amphibian diversity and species composition in relation to habitat type and alteration in the Mache-Chindul Reserve, northwest Ecuador. Herpetologica..

[10] Alcala AC, Bucol AA, Diesmos AC, Brown RM (2012). Vulnerability of Philippine amphibians to climate change. Philipp. J. Sci..

[11] Alcala EL, Alcala AC, Dolino CN (2004). Amphibians and reptiles in tropical rainforests fragments on Negros Island, the Philippines. Environ. Conserv..

[12] Herrmann HL, Babbitt KJ, Baber MJ, Congalton RG (2005). Effects of landscape characteristics on amphibian distribution in a forest-dominated landscape. Biol. Conserv..

[13] Olson DH, Anderson PD, Frissell CA, Welsh HH, Bradford DF (2007). Biodiversity management approaches for stream–riparian areas: perspectives for Pacific Northwest headwater forests, microclimates, and amphibians. Forest Ecol. Manag..

[14] Urbina-Cardona JN, Olivares-Pérez M, Reynoso VH (2006). Herpetofauna diversity and microenvironment correlates across a pasture–edge–interior ecotone in tropical rainforest fragments in the Los Tuxtlas Biosphere Reserve of Veracruz. Mexico. Biol. Conserv..

[15] Laurencio D, Fitzgerald LA (2010). Environmental correlates of herpetofaunal diversity in Costa Rica. J. Trop. Ecol..

[16] Russildi G, Arroyo-Rodríguez V, Hernández-Ordóñez O, Pineda E, Reynoso VH (2016). Species- and community-level responses to habitat spatial changes in fragmented rainforests: assessing compensatory dynamics in amphibians and reptiles. Biodivers. Conserv..

[17] Ficetola GF, Maiorano L (2016). Contrasting effects of temperature and precipitation change on amphibian phenology, abundance and performance. Oecologia.

[18] Brown RM (2013). The amphibians and reptiles of Luzon Island, Philippines, VIII: the herpetofauna of Cagayan and Isabela Provinces, northern Sierra Madre Mountain Range. ZooKeys..

[19] Sanguila MB (2016). The amphibians and reptiles of Mindanao Island, southern Philippines, II: the herpetofauna of northeast Mindanao and adjacent islands. ZooKeys..

[20] Siler CD (2011). Amphibians and reptiles, Luzon Island, Aurora Province and Aurora Memorial National Park, Northern Philippines: new island distribution records. Check List..

[21] Brown RM (2012). Amphibians and reptiles of Luzon Island (Philippines), VII: herpetofauna of Ilocos Norte Province, northern Cordillera Mountain Range. Check List..

[22] Siler CD (2012). Amphibians and reptiles, Romblon Island Group, central Philippines: comprehensive herpetofaunal inventory. Check List..

[23] Supsup CE, Guinto FM, Redoblado BR, Gomez RS (2017). Amphibians and reptiles from the Mt. Hamiguitan Range of eastern Mindanao Island, Philippines: new distribution records. Check List..

[24] Beukema W (2011). Herpetofauna of disturbed forest fragments on the lower Mt. Kitanglad Range, Mindanao Island. Philippines. Salamandra..

[25] Devan-Song A, Brown RM (2012). Amphibians and reptiles of Luzon Island, Philippines, VI: the herpetofauna of the Subic Bay area. Asian Herpetol. Res..

[26] Nuñeza OM, Ates FB, Alicante AA (2010). Distribution of endemic and threatened herpetofauna in Mt. Malindang, Mindanao. Philippines. Biodivers. Conserv..

[27] Relox RE, Leańo EP, Ates-Camino FB (2011). Herpetofaunal endemism and diversity in tropical forests of Mt. Hamiguitan in the Philippines. Herpetol. Conserv. Biol..

[28] (IUCN) International Union for Conservation of Nature. The IUCN Red List of Threatened Species. Version 2019-1, https://www.iucnredlist.org/species/ (2019).

[29] Vallan D (2002). Effects of anthropogenic environmental changes on amphibian diversity in the rain forests of eastern Madagascar. J. Trop. Ecol..

[30] Gillespie GR (2012). Conservation of amphibians in Borneo: relative value of secondary tropical forest and non-forest habitats. Biol. Conserv..

[31] Suazo-Ortuño I (2015). High resilience of herpetofaunal communities in a human-modified tropical dry forest landscape in western Mexico. Trop. Conserv. Sci..

[32] Riemann JC, Ndriantsoa SH, Raminosoa NR, Rödel M-O, Glos J (2015). The value of forest fragments for maintaining amphibian diversity in Madagascar. Biol. Conserv..

[33] Gardner TA (2007). The value of primary, secondary, and plantation forests for a neotropical herpetofauna. Conserv. Biol..

[34] Bickford D, Howard SD, Ng DJJ, Sheridan JA (2010). Impacts of climate change on the amphibians and reptiles of Southeast Asia. Biodivers. Conserv..

[35] Ndriantsoa SH, Riemann JC, Raminosoa N, Rödel M-O, Glos JS (2017). Amphibian diversity in the matrix of a fragmented landscape around Ranomafana in Madagascar depends on matrix quality. Trop. Conserv. Sci..

[36] Gillespie GR (2015). Responses of tropical forest herpetofauna to moderate anthropogenic disturbance and effects of natural habitat variation in Sulawesi. Indones. Biol. Conserv..

[37] Hillers A, Veith M, Rödel M-O (2008). Effects of forest fragmentation and habitat degradation on West African leaf-litter frogs. Conserv. Biol..

[38] Keller A, Rödel M-O, Linsenmair KE, Grafe TU (2009). The importance of environmental heterogeneity for species diversity and assemblage structure in Bornean stream frogs. J. Anim. Ecol..

[39] Wanger TC (2009). Conservation value of cacao agroforestry for amphibians and reptiles in South-East Asia: combining correlative models with follow-up field experiments. J. Appl. Ecol..

[40] Maynard RJ, Aall NC, Saenz D, Hamilton PS, Kwiatkowski MA (2016). Road-edge effects on herpetofauna in a lowland Amazonian rainforest. Trop. Conserv. Sci..

[41] Cortés-Gómez AM, Castro-Herrera F, Urbina-Cardona JN (2013). Small changes in vegetation structure create great changes in amphibian ensembles in the Colombian Pacific rainforest. Trop. Conserv. Sci..

[42] Díaz-García JM, Pineda E, López-Barrera F, Moreno CE (2017). Amphibian species and functional diversity as indicators of restoration success in tropical montane forest. Biodivers. Conserv..

[43] Suerte LO (2005). Geology, geochemistry and U-Pb SHRIMP age of the Tacloban Ophiolite Complex, Leyte Island (Central Philippines): Implications for the existence and extent of the Proto-Philippine Sea Plate. Resour. Geol..

[44] Corporal-Lodangco IL, Leslie LM (2017). Defining Philippine climate zones using surface and high-resolution satellite data. Proc. Comput. Sci..

[45] Tolentino PLM, Poortinga A, Kanamaru H, Keesstra S, Maroulis J, David CPC, Ritsema CJ (2016). Projected impact of climate change on hydrological regimes in the Philippines. PLoS ONE.

[46] Quiñones CMO, Asio VB (2015). Soils derived from ophiolitic rocks in northeastern Leyte: morphological, physical, and chemical properties. Ann. Trop. Res..

[47] Guimarães M (2014). One step forward: contrasting the effects of Toe clipping and PIT tagging on frog survival and recapture probability. Ecol. Evol..

[48] AmphibiaWeb. Available at https://amphibiaweb.org/ (2019).

[49] Madeira BG (2009). Changes in tree and liana communities along a successional gradient in a tropical dry forest in south-eastern Brazil. Plant Ecol..

[50] Kostel-Hughes F, Young TP, Carreiro MM (1998). Forest leaf litter quantity and seedling occurrence along an urban-rural gradient. Urban Ecosyst..

[51] Balaji D, Sreekar R, Rao S (2014). Drivers of reptile and amphibian assemblages outside the protected areas of Western Ghats. India. J. Nat. Conserv..

[52] Hammer Ø, Harper DAT, Ryan PD (2001). PAST: paleontological statistics software package for education and data analysis. Palaeontol. Electron..

[53] Gaspard G, Kim D, Chun Y (2019). Residual spatial autocorrelation in macroecological and biogeographical modeling: a review. J. Ecol. Environ..

[54] Paradis, E. et al. Package ‘ape’: analyses of phylogenetics and evolution. https://cran.r-project.org/web/packages/ape/ape.pdf (2019). 10.1093/bioinformatics/btg412 14734327

[55] Ranjitkar S, Xu J, Shrestha KK, Kindt R (2014). Ensemble forecast of climate suitability for the Trans-Himalayan Nyctaginaceae species. Ecol. Model..

[56] Oksanen, J. Vegan: an introduction to ordination. https://cran.r-project.org/web/packages/vegan/vignettes/intro-vegan.pdf (2019).

[57] Wood, S. Mgcv: mixed GAM computation vehicle with automatic smoothness estimation. https://cran.r-project.org/web/packages/mgcv/mgcv.pdf (2019).

[58] R Core Team. R: a language and environment for statistical computing. R Foundation for Statistical Computing, Vienna, Austria. https://www.Rproject.org/ (2019).

