# Supplementary material for: Impact of habitat alteration on amphibian diversity and species composition in a lowland tropical rainforest in Northeastern Leyte, Philippines
Source: Sci Rep. 2020 Jun 29;10:10547. doi: 10.1038/s41598-020-67512-6 (PMC7324599; doi:10.1038/s41598-020-67512-6)
Supplement: Supplementary file 1 — Supplementary file1 (PDF 513 kb) [file 41598_2020_67512_MOESM1_ESM.pdf]

Impact of Habitat Alteration on Amphibian Diversity and Species Composition in a Lowland Tropical Rainforest in Northeastern Leyte, Philippines

Syrus Cesar Pacle Decena*, Carlo Aguirre Avorque, Ian Christopher Pacle Decena, Pol Delbert Asis, Bryan Pacle

Environmental Management Department, Visayas State University-Alangalang, Alangalang, Leyte 6517, Philippines

**Corresponding Author: syrus_decenae2011@yahoo.com*

Supplementary Table S1: Correlation matrix of 8 environmental variables measured in stream strip plots in lowland tropical rainforest in northeastern Leyte, Philippines. USD = understorey density, T = temperature, E = Elevation, LLT = leaf litter thickness, LLV = leaf litter volume, DBH = diameter at breast height (1.3 m from the ground), TH = tree height, TD = tree density

Environmental Variables	USD	T	E	LLT	LLV	DBH	TH	TD
Understorey density	1							
Temperature	-0.50**	1						
Elevation	0.48**	-0.78**	1					
Leaf litter thickness	0.48**	-0.80**	0.86**	1				
Leaf litter volume	0.48**	-0.50**	0.71**	0.77**	1			
DBH	0.31	-0.65**	0.77**	0.67**	0.58**	1		
Tree height	0.44**	-0.73**	0.87**	0.84**	0.71**	0.88**	1	
Tree density	0.55**	-0.43**	0.51**	0.64**	0.71**	0.37*	0.58**	1

*$p<0.05$; **$p<0.01$

Supplementary Table S2: GAM models built with corresponding AIC values for abundance, richness and diversity of amphibians species (leaf litter and semi-aquatic species) in a lowland tropical rainforest in northeastern Leyte, Philippines. USD = understorey density, T = temperature, TD = tree density, DBH = diameter at breast height (1.3 m from the ground)

Model	AIC
Abundance	
1. Abundance ~ s(USD, k = 3) + TD + T + s(DBH, k = 3)	206.771
2. Abundance ~ TD + T + s(DBH, k = 3)	206.439
3. Abundance ~ T + s(DBH, k = 3)	206.185
Richness	
1. Richness ~ s(USD, k = 3) + TD + T + DBH	117.256
2. Richness ~ s(USD, k = 3) + TD + T	115.362
3. Richness ~ s(USD, k = 3) + TD	114.048
4. Richness ~ s(USD, k = 3)	112.818
Diversity	
1. Diversity ~ s(USD, k = 3) + s(TD, k = 3) + s(T, k = 3) + DBH	40.218
2. Diversity ~ s(USD, k = 3) + s(TD, k = 3) + s(T, k = 3)	38.570

Supplementary Table S3: Results of spatial autocorrelation test

**Variables**	**Moran's I**	***P* value**
Abundance	0.084	<0.001
Richness	0.082	<0.001
Diversity (Shannon-Wiener)	0.086	<0.001

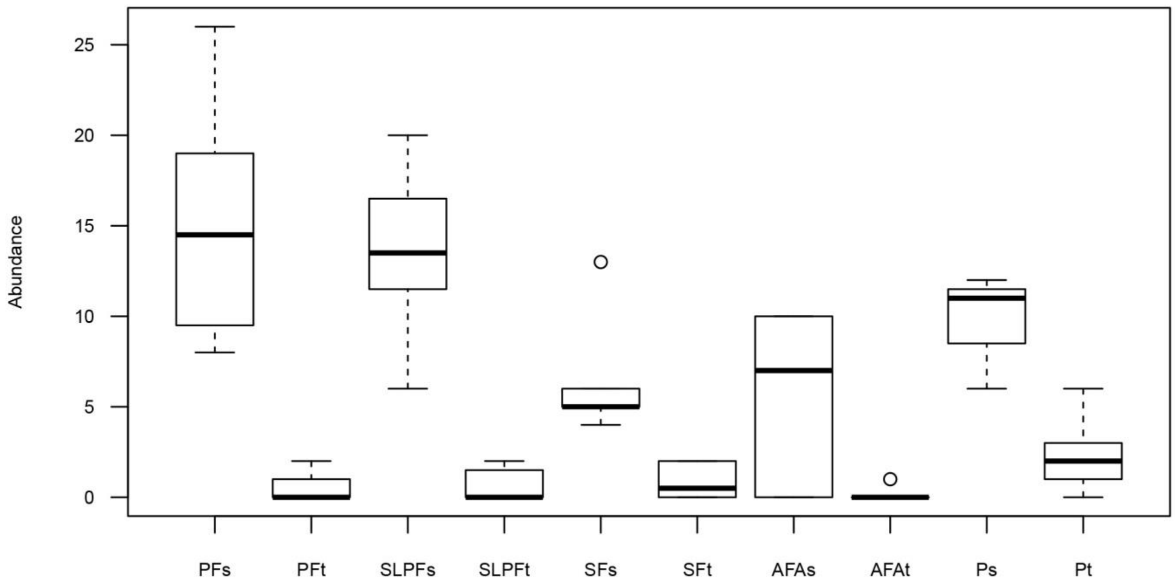

Supplementary Figure S1: Abundance of leaf-litter and semi-aquatic amphibians in stream (s) and terrestrial (t) strip plots between the different habitat alteration types in a lowland tropical rainforest in northeastern Leyte, Philippines. PF = primary forest, SLPF = selectively logged primary forest, SF = secondary forest, AFA = abandoned farm area, P = pasture. The central crossbars represent the median, the boxes the 75th and 25th percentile and the circles the outliers.

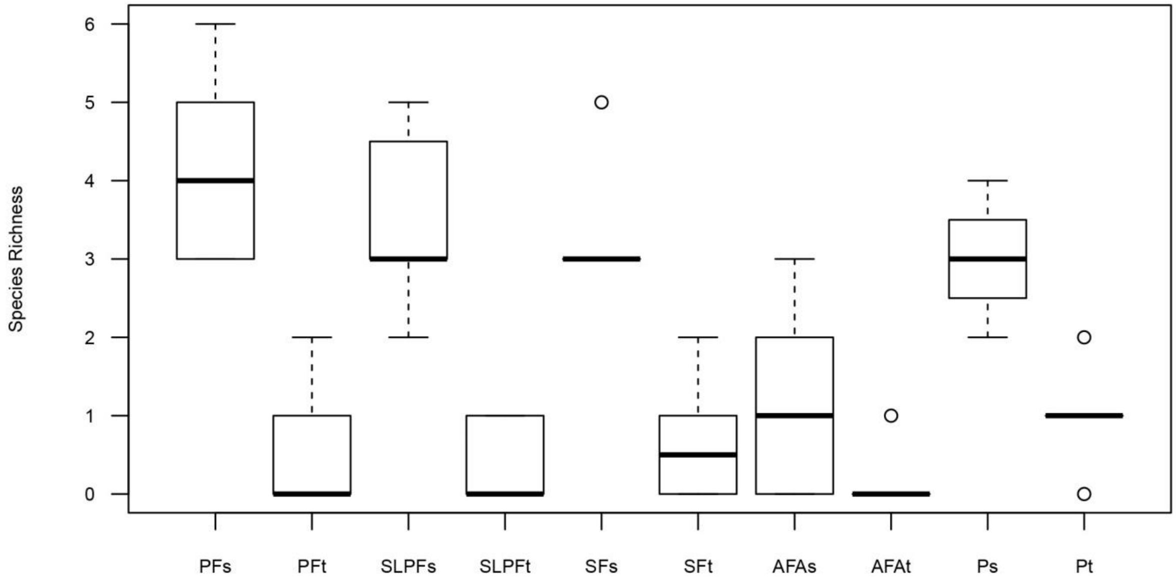

Supplementary Figure S2: Species richness of leaf-litter and semi-aquatic amphibians in stream (s) and terrestrial (t) strip plots between the different habitat alteration types in a lowland tropical rainforest in northeastern Leyte, Philippines. PF = primary forest, SLPF = selectively logged primary forest, SF = secondary forest, AFA = abandoned farm area, P = pasture. The central crossbars represent the median, the boxes the 75th and 25th percentile and the circles the outliers.

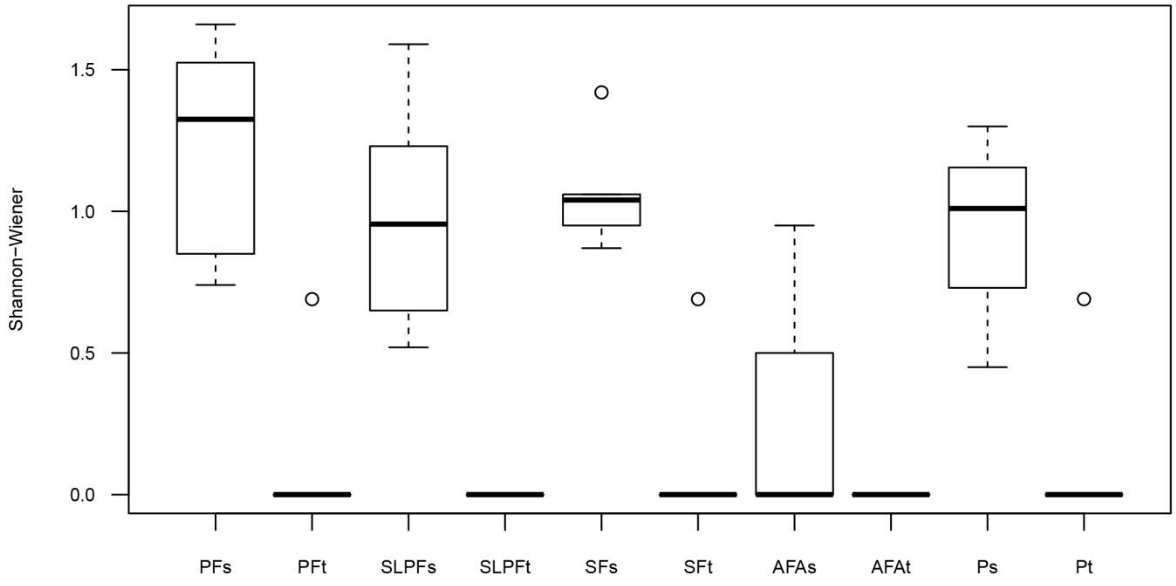

Supplementary Figure S3: Diversity (Shannon-Wiener) of leaf-litter and semi-aquatic amphibians in stream (s) and terrestrial (t) strip plots between the different habitat alteration types in a lowland tropical rainforest in northeastern Leyte, Philippines. PF = primary forest, SLPF = selectively logged primary forest, SF = secondary forest, AFA = abandoned farm area, P = pasture. The central crossbars represent the median, the boxes the 75th and 25th percentile and the circles the outliers.

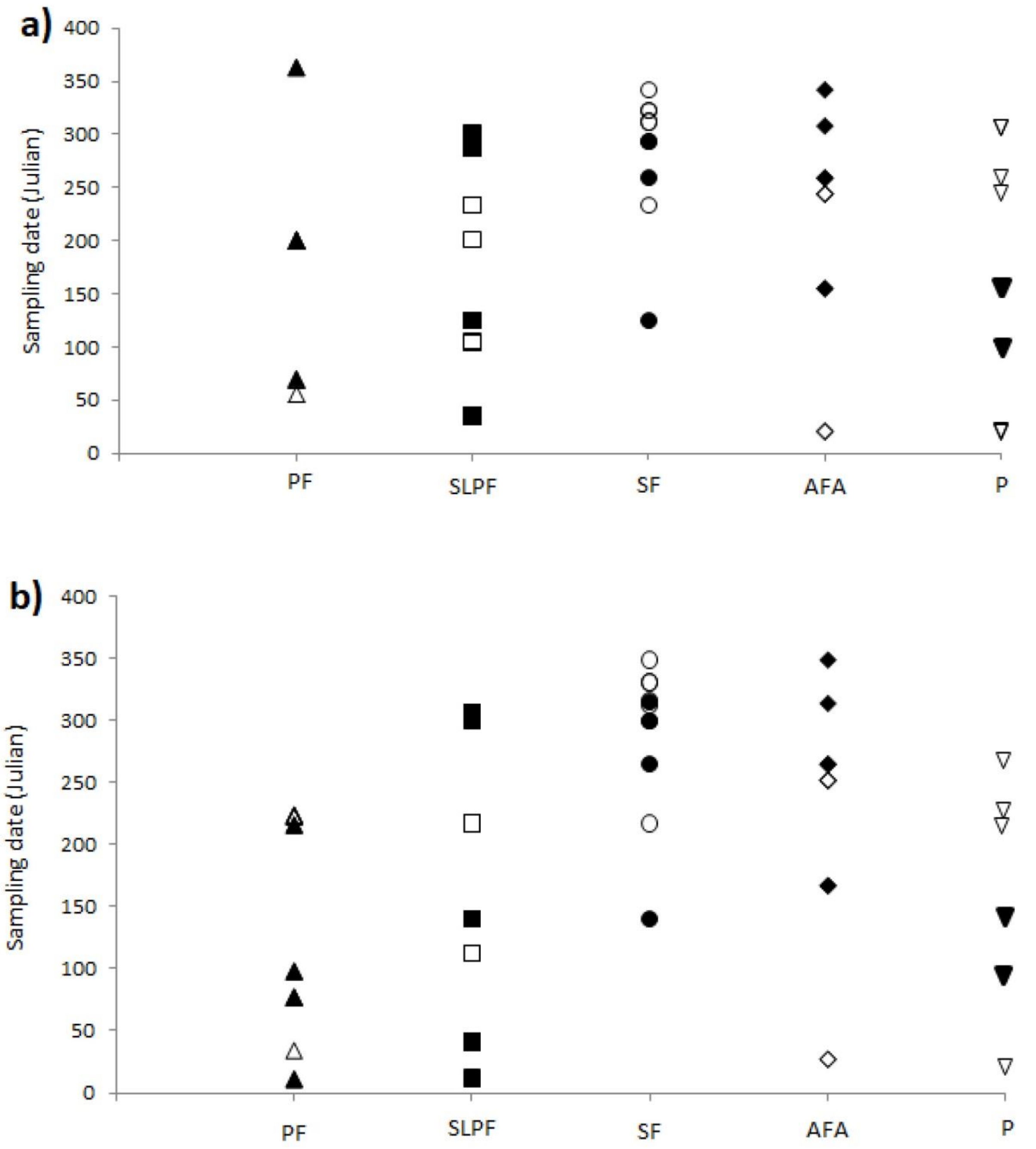

Supplementary Figure S4: The distribution of amphibian sampling sessions in each habitat alteration type during the **a)** first collection and **b)** second collection. PF = primary forest, SLPF = selectively logged primary forest, SF = secondary forest, AFA = abandoned farm area, P = pasture; solid symbols = stream, open symbols = terrestrial habitats.
